# Differential diagnosis of cognitive dysfunction in a multi-morbid patient

**DOI:** 10.1192/j.eurpsy.2023.2116

**Published:** 2023-07-19

**Authors:** S. Luna, S. González, R. J. Cortés, O. Kawas

**Affiliations:** 1 Psychiatry, Hospital Universitario “Dr. José Eleuterio González”, Monterrey, Mexico

## Abstract

**Introduction:**

Patients with systemic lupus erythematosus (SLE) have cognitive dysfunctions as a neuropsychiatric manifestation, associated with disabling symptoms. However, the presence of other medical or psychiatric comorbidities can delay or lead to a misdiagnose.

**Objectives:**

To present a case of a patient with diagnostic difficulty in the face of multiple medical and neurocognitive comorbidities.

**Methods:**

Description of a case report.

**Results:**

19-year-old female, Mexican, unemployed, with incomplete high school, with medical history of preterm birth by cesarean at 30 weeks due to placenta previa, history of early puberty, 4 years evolution of focal epilepsy, 1 year evolution of hypothyroidism and mild depression.

She began her symptoms 4 years ago, characterized by an abrupt onset of memory disturbances, decreased concentration, poor academic performance, infantile behavior, need for affection, alternated with irritability periods, verbal and physical aggression, repetitive and erratic behavior. She went to multiple specialists with different therapeutic approaches without clinical improvement. In 2020, she was referred to our service for evaluation, evidenciating a mild depressive episode and psychotherapeutic treatment was started.

Mental and neurological examination without alterations, normal vital signs, at physical examination: malar rash, oral ulcers, alopecia. Labs: increased erythrocyte sedimentation rate, normocytic anemia, leukopenia, rest normal. An electroencephalogram was requested, without alterations. Simple brain MRI was performed (Figure 1).

Psychological (figure 2) and Neuropsychological tests (table 1) were performed, showing alterations in memory recall and inhibitory control.

Due to the symptoms presented by the patient, SLE was suspected, and rheumatology evaluation was requested, integrating a diagnosis of incomplete SLE, and started treatment. The patient presented symptomatic improvement in cognitive symptoms and systemic signs. Likewise, a genetic evaluation was requested, without meeting the criteria for a genetic syndrome. The patient continues with symptomatic improvement and multidisciplinary treatment.

**Table:** 

**Total scores**	**Natural**	**Normalized**	**Diagnosis**
*Orbitomedial*	180	83	Mild alteration
*Pref-Anterior*	22	106	Normal
*Dorsolateral*	207	88	Normal
*BANFE total*	409	104	Normal

**Image:**

**Figure:**
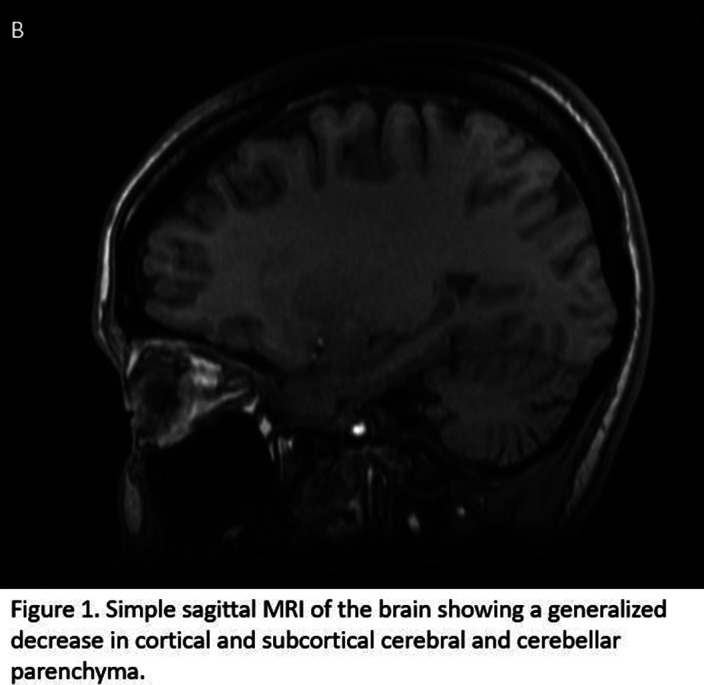

**Image 2:**

**Figure:**
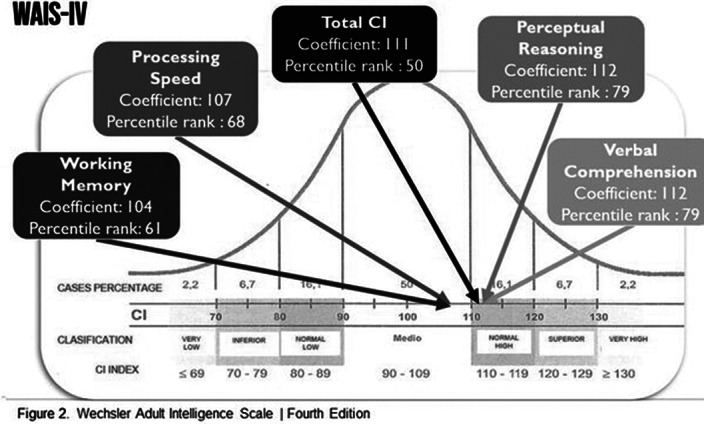

**Conclusions:**

Neurocognitive alterations are one of the most frequent manifestations of neurolupus, although its diagnosis and treatment may be delayed in the absence of clinical suspicion, mainly in multi-comorbid patients.

In the case, the patient presented multiple diseases that can explain a picture of neurocognitive impairment, such as epilepsy, depression, hypothyroidism. However, in these cases, a multidisciplinary approach is imperative, requiring to rule out the different causes of the patient’s symptoms.

**Disclosure of Interest:**

None Declared

